# Biomarkers for early diagnosis of AKI in the ICU: ready for prime time use at the bedside?

**DOI:** 10.1186/2110-5820-2-24

**Published:** 2012-07-02

**Authors:** Patrick M Honore, Rita Jacobs, Olivier Joannes-Boyau, Lies Verfaillie, Jouke De Regt, Viola Van Gorp, Elisabeth De Waele, Willem Boer, Vincent Collin, Herbert D Spapen

**Affiliations:** 1 Intensive Care Dept, Universitair Ziekenhuis Brussel, Vrije Universiteit Brussel, Brussels, Belgium; 2 Haut Leveque University Hospital of Bordeaux, University of Bordeaux 2, Pessac, France; 3 Department of Anaesthesiology and Critical Care Medicine, Ziekenhuis Oost-Limburg, Genk, Belgium; 4 Cliniques de l’Europe-Site St Michel, Brussels, Belgium; 5 Critical Care Nephrology Platform, Intensive Care Department, Universitair Ziekenhuis Brussel, Vrije Universiteit Brussel (VUB), 1090, Brussels, Belgium

**Keywords:** Hemofiltration, Biomarkers, Early diagnosis, Sepsis, Septic shock, SIRS, Acute kidney injury, Acute tubular apoptosis, Sepsis modulation, Blood purification, Dialysis, CRRT, Review

## Abstract

Because of its still rising incidence and high mortality rate in intensive care unit (ICU) patients, early recognition of acute kidney injury (AKI) remains a critical issue. Surprisingly, effective biomarkers for early detection and hence appropriate and timely therapy of AKI have not yet entered the clinical arena. We performed a systematic search of the literature published between 1999 and 2011 on potential early biomarkers for acute renal failure/kidney injury in an at-risk adult and pediatric population following the Quorum Guidelines. Based on this review, recommendations for the clinical use of these biomarkers were proposed. In general, kidney biomarkers may aid to direct early aggressive treatment strategies for AKI thereby decreasing the associated high mortality. To date, however, sensitivity and specificity of individual biomarker assays are low and do not sustain their routine clinical use. “Kits” containing a combination of established biomarkers, in conjunction with measured glomerular filtration rate, may enhance diagnostic and prognostic accuracy in the future.

## Review

### Introduction

Accurate diagnosis of AKI in an ICU setting is challenging. Indeed, the characteristic swings in renal function over time in critically ill patients largely reduce the validity of a sole creatinine-based AKI assessment. This has stimulated researchers to establish multidimensional classification systems that use specific criteria to grade AKI severity. At present, the RIFLE classification represents the most widely accepted tool to “score” AKI severity.

To better discriminate for AKI severity, early recognition of renal dysfunction, i.e., well before changes in serum creatinine do occur, has gained considerable interest. It has been evidenced that small or relative increases in serum creatinine are associated with a concomitant increase in patient mortality. However, even serial evaluation of serum creatinine levels does not allow to intercept adequately the rapidly evolving changes in renal function in severely ill subjects. Therefore, attention has focused on the development of “early” biomarkers, enabling diagnosis of AKI long before creatinine levels start to increase. Logically, a combination of biomarkers could enhance diagnostic accuracy, but validation of current individual candidate biomarkers is still underway.

### Looking for the “kidney troponin”

The intensive care community is faced with an important and frustrating dilemma. On the one hand, AKI is increasingly complicating the course of disease in critically ill patients causing several million deaths worldwide every year [1]. On the other hand, despite being aware of the AKI burden on outcome, ICU physicians remain deprived of effective biomarkers for early recognition of AKI. As a consequence, the delay in initiating appropriate therapy contributes significantly to the dismal prognosis of AKI [2].

In contrast with the variety of biological markers (including troponins) for early diagnosis of cardiac damage [3], standard assessment of renal function in ICU patients is still determined by assessment of serum creatinine levels and serum creatinine-based formulas [4]. However, while being designed primarily for longitudinal assessment of baseline renal function and validated merely in chronic kidney failure, creatinine-based decision-making in critically ill patients is debatable [4,5]. Accurate diagnosis of AKI becomes problematic when renal homeostasis is considerably influenced by rapidly evolving circulatory and inflammatory alterations. Under such conditions, the value of “classical” measures of renal function becomes doubtful.

Many studies have emphasized the role of AKI as an independent risk factor for mortality in the critically ill, causing patients to die from and not just “with” AKI [5,6]. This has stimulated the development of multidimensional AKI classification systems. Such classifications provide a more accurate description of AKI, allow AKI severity grading, and better predict outcome. At present, the most widely used classification is the RIFLE—an acronym for Risk, Injury, Failure, Loss, and End-stage renal disease—system, which was initially proposed by the Acute Dialysis Quality Initiative group [7] and has been validated in critically ill adult patients [8]. ICU patients with maximum RIFLE class R, I and F had hospital mortality rates of respectively 8.8%, 11.4%, and 26.3% [8].

It has been recently appreciated that vital body organs share information by virtue of various biological mediators. As a consequence, a pathology affecting one major organ can cause dysfunction of another apparently unrelated organ. The so-called cardiorenal syndrome, where primary dysfunction of the heart can cause kidney injury and *vice versa*, is a striking example of such organ crosstalk in critically ill patients. Because cellular injury occurs before clinical dysfunction, the potential of biological markers to detect subclinical molecular changes may render therapeutic interventions more effective. Extensive research is performed to find biomarker substances present in urine or serum, imaging techniques, or any other quantifiable parameters to detect—either alone or in combination—both heart and kidney dysfunction. Results of these investigations are still awaited [9].

### Why biomarkers should be preferred to creatinine: the mortality issue

Small or relative increases in serum creatinine are associated with increased patient morbidity and mortality independently of expected mortality as calculated by mortality risk scores. Chertow and colleagues found a nearly sevenfold increase in the odds of death for patients presenting a 0.5 mg/dL increase in serum creatinine, even when adjusted for numerous comorbidities [6]. As stated previously, serum creatinine concentrations as well as creatinine clearance are unreliable indicators of acute or abrupt changes in kidney function in ICU patients [10]. Serum creatinine comes into play as a marker for decreasing kidney function when already more than 50% of kidney function has been lost and is only useful after a steady state has been reached. The latter can take time (sometimes up to 48 h), especially in ICU patients [11]. Creatinine clearance is a better marker for changes in kidney function, although again with considerable delay because of the time lag between cellular kidney damage and loss of kidney function [12]. Studies in humans have shown that AKI can be prevented or more adequately contained if treatment is instituted shortly after the initial kidney insult [13]. This underscores the need for a biomarker to early detect kidney injury in the ICU [14].

Apart from allowing a more early diagnosis of kidney damage, biomarkers also may be used to better differentiate the etiology of AKI, resulting in more appropriate or adjusted treatment [15]. The potential benefits of such approach are immense as the presence of AKI enhances the intrinsic mortality caused by the initial and/or underlying disease [16,17]. Furthermore, epidemiologic studies have shown that half of the cases of AKI in the ICU are related to sepsis, with a superimposed mortality that is higher than in nonseptic AKI [18].

### Assessing specificity and sensitivity of biomarkers using creatinine-based criteria: a flawed paradigm?

Diagnostic specificity and sensitivity of biomarkers are currently weighed against creatinine-based criteria. Such comparative relationship is questionable, because creatinine itself is a rather poor biomarker of renal function. As a consequence, increased levels of one specific biomarker in the presence of normal creatinine values may point to true renal injury (which creatinine fails to detect) or be falsely positive. As outlined by Waikar et al. [19], creatinine levels by no means reliably reflect renal function. Creatinine definitely is no marker of kidney injury and merely a surrogate for evaluation of renal function . In the acute critical care setting, the role of creatinine as a marker of kidney injury may even be more criticized. For example, the syndrome “pre-renal azotaemia” may biochemically (i.e., by changes in creatinine concentrations) resemble “acute tubular necrosis.” However, the underlying pathophysiology, treatment implications and prognosis of these disorders are markedly different. Intensive care physicians familiar with AKI know that creatinine can be deceptively normal when kidneys are failing [19]. In a sense, we are on the verge of a potential paradigm shift in critical care nephrology, where creatinine may be replaced by novel biomarkers for diagnosis of tubular injury. Various biologically plausible biomarkers have indeed been identified in animal models and, even if potentially imperfect, await validation in the clinical arena [20]. It is hoped that future diagnosis of AKI in critically ill patients will be freed from the creatinine “stigma” in the way that diagnosis of myocardial infarction exchanged measurement of lactate dehydrogenase and creatine phosphokinase for the highly specific troponin recording.

### Potential biomarker candidates

Actually, more than ten promising biomarkers for AKI have been identified. The most relevant substances are neutrophil gelatinase-associated lipocalin (NGAL) [21], cystatin C [22], kidney injury molecule-1 (KIM-1) [23], beta-2 microglobulin (β_2_M) [24], and interleukin-18 (IL-18) [25].

NGAL is a 25-kDa protein bound to gelatinase from neutrophils [26]. In animal models, NGAL appears immediately after induction of an ischemic or nephrotoxic insult [26,27]. Devarajan and coworkers recently completed remarkable downstream proteomic analyses on NGAL [28]. Preclinical transcriptome profiling studies in acutely injured animals identified the NGAL gene as one of the most and very early upregulated ones expressed in the kidney [29]. Additional analyses in animals confirmed that NGAL was amongst the most intensely induced proteins in the kidney after ischemic or nephrotoxic injury [30].

Plasma and urinary NGAL have shown promise as early biomarkers of clinical AKI in cardiopulmonary bypass surgery, kidney transplantation, following intravenous contrast administration, and in ICU patients [25-29]. NGAL measurement has been employed as an outcome variable in clinical trials that investigated the effect of hydroxyethylstarch infusion over other colloids on maintaining renal function in elderly cardiac surgery patients [31-33]. Also, an attenuated response of urinary NGAL was found in adult cardiac surgery patients in whom a lower incidence of AKI was noted when sodium bicarbonate was administered instead of sodium chloride [34]. Notwithstanding these promising results, the number of patients studied remains small and a large, prospective, multicentric study is needed to delineate the exact role and potential of NGAL [28,35].

Cystatin C is a protease inhibitor that is released into the blood, filtered through the glomerulus, and completely reabsorbed in the proximal tubule. In chronic kidney disease, cystatin C was found to better predict glomerular function than serum creatinine [36]. In cardiac surgery patients, a 50% increase in cystatin C could predict AKI 48 h before changes in serum creatinine or creatinine clearance suggested renal dysfunction [37]. In practice, however, NGAL is still an earlier biomarker than cystatin C [37].

KIM-1 was identified as a transmembrane protein produced by the proximal tubule following ischemic or toxin-induced AKI [23]. KIM-1 is measured in the urine. It has been thoroughly investigated in patients undergoing cardiopulmonary bypass where it proved to be more specific than NGAL in detecting ischemic and nephrotoxic AKI [23]. In this particular setting, KIM-1 adds specificity whilst NGAL proved to be more sensitive. Cystatin C, while also being a very sensitive marker, was less specific as compared to KIM-1 and NGAL [5,38].

β_2_ -microglobulins are low-molecular-weight proteins freely filtered by the glomerulus [39]. Unlike creatinine, their serum concentrations are less dependent on extrarenal factors [39]. In a recent cross-sectional analysis, Herrero-Morin and coworkers showed that cystatin C and β_2_M was found to be largely superior to a creatinine clearance cutoff <80 mL/min/1.73 m^2^ for detecting decreased glomerular filtration rate in critically ill children [24].

IL-18 is a proinflammatory cytokine that is produced at the proximal tubular level. IL-18 has been identified as a very early biomarker of AKI in kidney transplant patients, acute respiratory distress syndrome, and after cardiopulmonary bypass surgery [40].

A major drawback limiting the routine use of biomarkers is their low individual sensitivity and specificity, generally not exceeding 70–75%, for detecting early kidney damage [41]. One possible solution might be the creation of a kit incorporating various biomarkers to allow quick and accurate diagnosis of AKI while patients are still in the ICU [5,42,43]. As therapy (e.g., CVVH) may then not only be started more early but also becomes tailored to the type of AKI, a huge impact on outcome might be expected [44].

### Synergy between biomarkers and serum creatinine

The potential synergy of concomitant use of biomarkers and serum creatinine-derived measurement of glomerular filtration rate (GFR) must be underlined. In a recent study, NGAL and serum creatinine-derived GFR, measured at ICU admission, both predicted development of severe AKI. However, the combination of NGAL and GFR assessment significantly increased predictive accuracy. Interestingly, in the presence of normal serum creatinine values, NGAL alone remained predictive for AKI [45].

### Conclusions and recommendations

Early diagnosis of AKI, particularly in severely ill patients, remains difficult. Creatinine-based measurements prove to be little helpful. Serum creatinine levels start to increase when most of the kidney function is already lost and a steady state has been reached. Creatinine clearance also remains a late and indirect marker of AKI [46-48].

Currently, much attention goes to biomarkers that are able to detect AKI in an earlier phase of development. Most promising are NGAL, cystatin C, KIM-1, β_2_M, and IL-18. Apart from a role in diagnosis, some markers (e.g., NGAL) also may have benefit in assessing eventual unwarranted renal effects of toxins, ischemic events, and infusion fluids. Validation of those kidney markers in various conditions of AKI is actually ongoing [47,48]. However, clinical studies on biomarkers are still scarce, particularly in critically ill patients, and sensitivity and specificity of individual biomarkers remain unacceptably low [48]. Development of biomarker kits that combine markers with different characteristics may increase diagnostic accuracy. Large multicentric randomized studies are imperative to confirm whether the use of biomarkers can influence course and treatment of AKI [49,50]. Finally, linking the assessment of biomarker specificity and sensitivity to creatinine-based criteria should be abandoned. An early and goal-directed therapy for AKI by using offensive strategies that are “covered” and directed by early and serial evaluation of biomarkers probably is the ultimate method to combat excessive “renal” mortality in the ICU.

## Competing interests

The authors declare that they have no competing interests.

## Authors’ contributions

PMH, RJ, OJB, and HDS conceived and wrote the review. LV, WB, JDR, EDW, VC and VVG participated in literature search and selected appropriate articles. PMH and HDS participated in design, coordination, and writing. All authors read and approved the final manuscript.
